# A microfluidic approach to rapid sperm recovery from heterogeneous cell suspensions

**DOI:** 10.1038/s41598-021-87046-9

**Published:** 2021-04-12

**Authors:** Steven A. Vasilescu, Shayan Khorsandi, Lin Ding, Sajad Razavi Bazaz, Reza Nosrati, Debra Gook, Majid Ebrahimi Warkiani

**Affiliations:** 1 School of Biomedical Engineering, University of Technology Sydney, Sydney, NSW 2007 Australia; 2 Department of Mechanical and Aerospace Engineering, Monash University, Clayton, VIC 3800 Australia; 3 Institute for Biomedical Materials and Devices (IBMD), Faculty of Science, University of Technology Sydney, Sydney, NSW 2007 Australia; 4 Reproductive Services, Royal Women’s Hospital/Melbourne IVF, Melbourne, Australia; 5 Department of Obstetrics and Gynaecology, University of Melbourne Victoria, Melbourne, Australia

**Keywords:** Biotechnology, Urology, Engineering, Physics

## Abstract

The isolation of sperm cells from background cell populations and debris is an essential step in all assisted reproductive technologies. Conventional techniques for sperm recovery from testicular sperm extractions stagnate at the sample processing stage, where it can take several hours to identify viable sperm from a background of collateral cells such as white bloods cells (WBCs), red blood cells (RBCs), epithelial cells (ECs) and in some cases cancer cells. Manual identification of sperm from contaminating cells and debris is a tedious and time-consuming operation that can be suitably addressed through inertial microfluidics. Microfluidics has proven an effective technology for high-quality sperm selection based on motility. However, motility-based selection methods cannot cater for viable, non-motile sperm often present in testicular or epididymal sperm extractions and aspirations. This study demonstrates the use of a 3D printed inertial microfluidic device for the separation of sperm cells from a mixed suspension of WBCs, RBCs, ECs, and leukemic cancer cells. This technology presents a 36-fold time improvement for the recovery of sperm cells (> 96%) by separating sperm, RBCS, WBCs, ECs and cancer cells into tight bands in less than 5 min. Furthermore, microfluidic processing of sperm has no impact on sperm parameters; vitality, motility, morphology, or DNA fragmentation of sperm. Applying inertial microfluidics for non-motile sperm recovery can greatly improve the current processing procedure of testicular sperm extractions, simplifying the fertility outcomes for severe forms of male infertility that warrant the surgery.

## Introduction

With the rising trend of infertility worldwide, 15% of all couples fail to naturally conceive a child, even after 1 year of consistent sexual activity. Male infertility is the sole cause of 30% of infertility cases and a contributing factor 50% of the time^1^. While assisted reproductive technology (ART) has proven effective in assisting infertile couples in their reproductive challenges, there still exist several sub-types of male infertility that remain exceedingly difficult to treat successfully with ART. Azoospermia, accounting for 10–20% of infertile men and 1% of the general male population, is the most severe form of infertility, defined as the absence of spermatozoa in centrifuged semen on at least two occasions^2,3^. In some cases, this is reversible and may be treated through the use of hormonal therapy; however, many cases require surgical extraction of testicular tissue in order to attain viable sperm for use in ART^4^.

Generally, azoospermia can be classified into Obstructive Azoospermia (OA) and Non-Obstructive Azoospermia (NOA), consisting of 60% of azoospermia cases^5^. OA defines as when sperm production is normal within the seminiferous tubules, but no sperm are present in the semen after ejaculation due to obstruction of the reproductive tract. On the other hand, NOA results from compromised sperm production. While OA can be treated through surgical reconstruction of the reproductive tract, treatment of patients with NOA is more challenging^6^. For those suffering from NOA, treatment typically consists of surgical extraction of viable sperm from the testis or epididymis, followed by intracytoplasmic sperm injection (ICSI). The two most common surgical approaches taken in use are Microdissection Testicular Sperm Extraction (mTESE) and Testicular Sperm Aspiration (TESA).

mTESE, is the more preferred method for NOA. In this procedure, a testis is revealed through a scrotal incision then bi-valved exposing the seminiferous tubules. Through careful analysis under a microscope, a dilated tubule likely to contain sperm is identified and extracted from the testicle. Once the harvested tubule is confirmed to potentially contain sperm, the testis is sewed back in place^4^. mTESE is reported to have a high sperm retrieval rate of up to 64%, which is far higher than other methods. Specifically, mTESE has shown to be 17% more effective than conventional TESE, with conventional TESE being 28% more effective than TESA^7^. However, biopsied and aspirated testicular sample processing is a time-consuming procedure that requires expertise and precise equipment and is subjected to human error and fatigue^8^. Tissue extracted from a testicular biopsy will consist of not only sperm, but also RBCs, WBCs, and epithelial nurse cells such as Sertoli and Leydig cells, as well as cellular debris. This combination can be further complicated by the presence of blood borne malignancies (leukaemia) which have infiltrated the site of sperm production such as in peri-pubertal males with Leukaemia, where fertility preservation before chemotherapy is a priority^9,10^. Of those diagnosed with Leukaemia, 90% of men freeze their sperm; however, a danger arises with the possibility of the frozen sperm being contaminated with leukaemia cells^11^. Biopsied tissue is typically minced, and suspended in sperm media to enable microscopic examination and identification of sperm cells. Morphologically assessed sperm cells are then manually extracted for use in ICSI. This manual examination process is performed by a trained professional and typically takes 2–4 h, but can take up to 14 h depending on the amount of sperm and the level of contamination present in the sample^8,12^. Some downstream purification techniques include introducing an erythrocyte lysing buffer to remove red blood cells or using pentoxifylline to induce motility in sperm. While these techniques are somewhat effective, they do affect sperm viability and compound the time and steps already present in the ART process, and therefore, are not ideal^13^.

This whole examination process is long and tedious and greatly reliant on the skill and experience of the examiner. Viable sperm are easily missed due to many variables such as inexperience, exhaustion, and cell density, resulting in a process that is prone to human error^14^. For patients with NOA, who already have only a few sperm in their sample, if sperm are overlooked due to human error, this could wrongly indicate absolute infertility^15^. In addition, extended periods of exposure during the examination process may negatively impact cell viability and therefore fertilization potential^16^. As such, a more efficient and higher throughput method, able to locate and isolate sperm from the suspension would greatly benefit the clinical workflow assisting severe forms of male infertility.

Many studies have attempted to provide autonomous and rapid cell isolation, but perhaps the most exemplary technology which continues to deliver innovative solutions to cell separation is microfluidics^17^. Microfluidics has proven to be effective in the selective isolation of inorganic and organic microparticles from suspension, such as circulating tumour cells from blood or urine^18,19^. Techniques such as pinched flow fractionation or microfluidic di-electrophoresis have been applied as sperm selection tools with encouraging results. However, these techniques are associated with limitations that impede their clinical translation. Pinched flow fractionation is heavily reliant on the shape of cells at a small junction which may limit its application for non-motile sperm isolation. It also provides less predictable trajectories for cells such as RBCs^20^, which have a disc-like shape and represent a major proportion of the background cell population in testicular biopsies. Alternatively, more active methods such as dielectrophoretic cell sorting, which apply an external electrical field to mediate cell sorting, have been presented for non-motile sperm sorting^21^. While potentially effective, these types of methods are typically expensive, inherently complex, and struggle to accommodate large concentrations of highly heterogeneous sample populations. Furthermore, these devices are typically fabricated using conventional microfluidic materials and methods such as PDMS and soft photolithography, respectively, which are not amenable to rapid idea generation and testing.

Microfluidic technologies have already shown promising opportunities for male infertility treatment and ART over the past two decades, with the vast majority of technologies targeting motile sperm populations^22,23^. Motility-based sperm selection devices have employed laminar flow boundaries, chemotaxis, and rheotaxis to achieve effective sperm selection^24–28^. These, and other microfluidic sperm separation approaches, are heavily reliant on sperm motility as a fundamental mechanism for sperm selection, with disproportionately fewer studies focusing on the isolation of non-motile sperm from background cell contaminants^8^. For men who undergo testicular surgery due to azoospermia, we have developed a microfluidic, 3D printed sperm separation device. This technology allows technicians to recover sperm from biopsied tissue suspensions within 5 min, effectively eliminating the human error associated with extended sample microscopy. We demonstrate that this selection process preserves the vitality, motility, morphology, and DNA integrity of the sperm while simultaneously reducing the effective labour time to find sperm in suspension from hours to minutes. Furthermore, by limiting their time in vitro and their exposure to reactive oxygen, digestive enzymes, and cellular debris, this method reduces the potential for harmful sperm exposure and allows technicians to distribute their attention to other valuable tasks in the ART workflow. This setup may also prove useful in the isolation of sperm and or spermatogonial stem cells from peri and prepubertal leukemic males for fertility preservation or in the purification of semen samples presenting pyospermia (high white cell contamination in semen).

## Materials and methods

### Device fabrication

Devices were fabricated using modified additive manufacturing techniques previously reported by our group for inertial microfluidic devices^29,30^. 3D printing was performed using a high-resolution Digital Light Processing (DLP) 3D printer (MiiCraft, Hsinchu Taiwan). The desired geometry was drawn in SolidWorks 2018 × 64 Premium Edition and then exported as an STL file to Miicraft software (MiiCraft 125, Version 4.01, MiiCraft ), for pre-processing of the printing options. Once the print options were confirmed, the part was set to print.

The microfluidic device used in this study had a trapezoidal cross-section with 30 and 90 µm wall heights connected by a 300um width base. After printing, the chip was thoroughly washed with Isopropanol Alcohol (IPA) and DI water (three times). Between each wash, the part was blow-dried with a pressurized air gun, making sure all residual liquid resin was removed. The part was then cured under ultraviolet (UV) light for 60 s in 20-s increments, turning the part between each increment. It is worth noting that excessive UV treatment may result in the formation of stress fractures.

Once the chip is ready, it was then attached to Poly-methyl methacrylate (PMMA) sheet using a transparent double-sided pressure-sensitive adhesive tape (ARcare, Adhesive Research) coated with AS-110 acrylic medical grade adhesive was cut, compressed with a bench clamp to promote strong bonding. This approach effectively binds open 3D-printed microchannels with optically transparent acrylic sheets, producing a tightly sealed microchannel. Our previous study has confirmed that the bond strength of this method is appropriate for high pressure inertial microfluidic particle separation^30^.

### Microfluidic device characterization

In order to characterise the devices performance before human sperm sampling, solutions of fluorescent polystyrene microbeads (Fluoresbrite Microspheres, Polysciences, Singapore) were loaded into two plastic syringes (BD Bioscience) with Luer-lok tips and pumped through the device at a range of flow rates (0.5–1.5 mL/min) to observe particle focusing behaviour. Samples were pumped through tygon tubing (inner diameter 0.508 mm, outer diameter 1.524 mm) using a Fusion Touch Chemyx syringe pump (Fusion 200, Chemyx, North America). Fluorescent microbead solutions were prepared with 0.01% volume fraction of particles added to the MACS buffer (Miltenyi Biotech, Australia). Particle diameters of 3.2, 5, 9.8, and 20 µm were tested and representatives of the cell types to be used, Sperm, RBCs, WBCs, and cancer cells, respectively. The primary usage of MACS buffer is to prevent nonspecific adhesion of microbeads to the tubing of the microchannel. The distribution of particles was recorded using an Olympus Ix73 inverted microscope with an Olympus DP80 camera for fluorescent imaging in the GFP channel.

### Cell culture

K562 cells (chronic myeloid leukaemia cells, PHE Culture Collections, UK) were cultured in cell culture media containing RPMI with 10% FBS and 1% Penicillin streptomycin at 37 °C and 5% CO_2_ until confluent. Since this cell line is non-adherent, one third of the total flask volume was removed and centrifuged at 500 RCF for 5 min and the supernatant replaced with 1 mL of Sperm Rinse media (Vitrolife). The remaining cells were also centrifuged and washed with cell culture media before being placed in a new flask.

### Human cell preparation

Human semen samples were obtained through ejaculation after 2–5 days of sexual abstinence (as recommended by the World Health Organisation^31^). Raw semen samples were incubated at 37 °C for 30 min to allow for full liquefaction. The sample is then placed in a centrifuge (Heraeus Multifuge X1) for 8 min at 800 RCF to separate the sperm pellet from the seminal plasma. Standard semen parameters were obtained in accordance to WHO guidelines^32^. All donors signed an informed consent, and this study was approved by the ethics review board at UTS (ETH19-3677).

RBC samples were obtained from whole blood specimens within 3 days of collection. Collected blood samples were also resuspended in Sperm Rinse (SR) media. Two sets of mixed cell suspensions were created for microfluidic sperm recovery: a solution of sperm, RBCs, WBCS and epithelial cells, and a solution of sperm with K562 cells. All cells were mixed in warm SR media. Raw semen samples were diluted down to between 1 × 10^5^ and 1 × 10^6^ cells/ml, RBC concentration was roughly 8 × 10^6^ cells/mL (approximated number for a TESE sample), WBCs (purchased from IQ Biosciences, 10 × 10^6^ cells/mL) were diluted to a concentration between 5 × 10^5^ and 1 × 10^6^ cells/mL, and ECs were diluted to a concentration of between 7 × 10^5^ and 1 × 10^6^ cells/mL. Epithelial cells were isolated from 3 donors with high concentrations of background cell populations and cryopreserved until needed. To better mimic pyospermic semen samples, 1 mL of the raw semen sample was spiked with the same concentrations of WBCs before microfluidic sorting. Spiked semen samples were run through the microfluidic device raw and after being washed via centrifugation (8 min at 800 RCF) twice. Sperm and K562 cell solutions contained the same number of sperm cells per mL while cultured K562 cells were centrifuged at 4500 RCF for 5 min, cell culture supernatant removed and resuspended in SR media. The final concentration of K562 cells was 1 × 10^6^ cells/mL.

In this study, cell separation experiments were repeated up to ten times and the efficiency of separation presented as the average percentage separation with standard deviation. Experiments assessing sperm quality metrics (vitality, motility, morphology and DNA integrity) were repeated three times each.

### Device operation

The microfluidic device was initially pre-wet with SR media and any bubbles removed by manually injecting fresh SR media into the device. Using two 5 mL plastic syringes, the mixed solution of sperm, red blood cells (RBC), and white blood cells (WBC) were injected into the device at its inlets. Both syringes infuse the solution at flow rates of 0.5, 0.55, and 0.6 mL/min, resulting in combined flow rates of 1, 1.1, and 1.2 mL/min. These flow rates were chosen based on the focusing behaviour observed in microparticle experiments. Mixed cell suspensions were injected via two inlets to reduce the overall pressure on the chip’s inlet. Cell suspensions pumped through the chip were collected from two separate outlets in 1.5 mL graduated microtubes. Human sperm concentration from each outlet was measured using a hemocytometer and the counts used to determine the separation efficiency at the tested flow rates.

### Sperm vitality, morphology, and motility analysis using open CASA

To assess the impact of the chip on sperm populations human sperm vitality, morphology and motility were assessed on sperm samples before and after microfluidic processing. Collected sperm samples from both outlets were then immediately combined and re-cycled into the chip up to four times. After each cycle, a 100 µL aliquot of sperm cell suspension was taken for analysis. In each test, 2 mL of the sperm sample with 1 × 10^6^ sperm per mL in concentration was run in its entirety at 1.1 mL/min.

Vitality was assessed using the fluorescent-based LIVE/DEAD sperm viability kit (ThermoFisher Scientific), by staining live and dead sperm according to the supplier manual. Collected fluid fractions were loaded into a hemocytometer and observed using an Olympus Ix73 inverted microscope with an Olympus DP80 camera for fluorescent imaging. Cells with green fluorescence (live cells) and red fluorescence (dead cells) were counted using the cell counter plugin from ImageJ (bundled with Java 1.8.0), and the ratio of live cells to total cells was used to quantify sperm vitality in the sample.

In addition, sperm motility was evaluated by taking 15 s videos of sperm swimming in bulk SR medium, immediately before and after microfluidic sample processing. The videos were analyzed using the openCASA plugin in ImageJ (version 1.0) to obtain sperm motility parameters^32^. Motility parameters were set to be compatible with previously reported CASA systems^33,34^. After obtaining the motility data from the CASA plugin for ImageJ, the sperm trajectories were visually cross-referenced to ensure the accuracy of the data collected.

Morphology was assessed by Diff-Quik stain kit (Microptic SL) using strict criteria according to Kruger *et al*^34^. Following the collection of the sorted fluid fractions, a concentration-dependent droplet of the sperm suspension was smeared across a glass slide and air-dried at room temperature. The dried slides were then stained with the components from the Diff-Quik Stain Kit. Following this protocol, resulted in minimal background staining and good colour contrasts. Abnormal cells were determined by having any one of a number of specific abnormalities (i.e. amorphous, round, midpiece defect, thin, elongated etc.). A total of 100 sperm were assessed per cycle through the microfluidic device.

### Sperm DNA Analysis

Assessment of DNA fragmentation index (DFI) was performed by a modified sperm chromatin dispersion (SCD) test, using the HT-HSG2 kit (Halotech DNA). The DFI of sperm was obtained before and after each of four microfluidic circulations of the sperm sample through the spiral. To create the sperm suspensions, sperm were washed by centrifugation as previously mentioned and suspended in SR to a concentration of 1 × 10^6^ sperm per mL in a 2 mL total volume. Samples were tested for DFI immediately before microfluidic processing of the solution as well as immediately after one, two, three and four passes through the device. The sample was run in its entirety at 1.1 mL/min and the collected sperm were aliquoted for SCD assay. To perform the SCD assay, 90 μL of semen suspension was added to an Eppendorf tube and mixed with pre-warmed agarose. 10 μL of the semen-agarose mixture was pipetted onto super-coated slides and covered with a coverslip. The slides were placed on a cold plate at 4 °C for 5 min to allow the agarose set. The coverslips were gently slid off the slides, and the slides immediately immersed horizontally in an acid solution (from the kit) and incubated for 7 min. The slides were then gently tilted vertically to allow the acid solution to run off the slides. The slides were horizontally immersed in 10 mL of the lysing solution for 20 min, then washed with distilled water for 5 min. The slides were then dehydrated in increasing concentrations of ethanol (70%, 90%, and 100%) for 2 min each, air-dried, and stored at room temperature in the dark.

For bright-field microscopy, slides were horizontally covered with a mix of Wright's staining solution (Merck) and phosphate-buffered solution (1:1; Merck) for 5 min. Then the slides were briefly washed in DI water. A minimum of 300 spermatozoa per sample were scored.

SCD analysis was performed by counting the number of sperm with and without visible halos as per the test manufacturer’s instructions. Sperm cells without a halo or with a weakly stained, small or degraded halo were considered to have fragmented DNA, while sperm cells with medium to large halos were considered to have intact DNA. DFI is expressed as the percentage of sperm cells with fragmented DNA.

## Results and discussion

**Figure 1:**
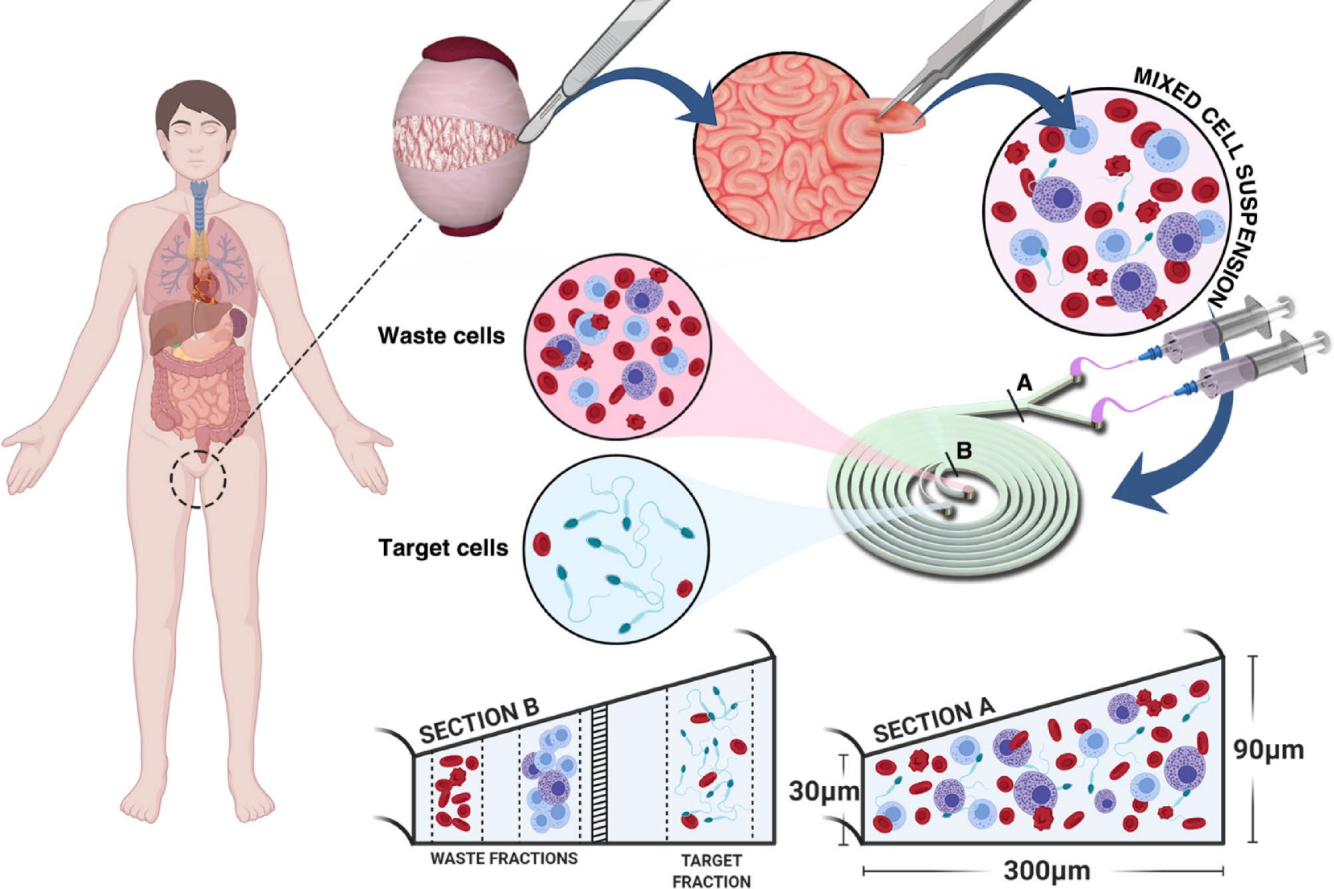
Schematic illustration of how microfluidics can be implemented during the sample processing stage of biopsied testicular tissue operations. Section A and B Illustrate the representative internal focusing positions of cells at the inlet and outlet of the device respectively.

We have developed a 3D printed inertial microfluidic device as a viable alternative for sperm isolation from mixed cell suspensions. Inertial microfluidics exploits the interplay of focusing forces present within spiral microchannels, leveraging the variability in size and deformability of different cell types to perform high throughout cell separation. The microfluidic separation device used in this study was a 3D printed spiral microchannel, which was developed using previously reported fabrication methods^30,35,36^. This 3D printed microfluidic device not only allows for high throughout and efficient sperm recovery, but also presents new focusing behaviour for cells of a certain size, not seen in flexible PDMS microchannels. Figure 1 outlines the representative workflow of how a microfluidic setup could be inserted into a clinical sperm isolation procedure. Sections A and B show the distribution of different cell populations within our 3D printed microfluidic spiral at the inlet and outlet of our device, respectively.

### Inertial focusing behaviour

**Figure 2:**
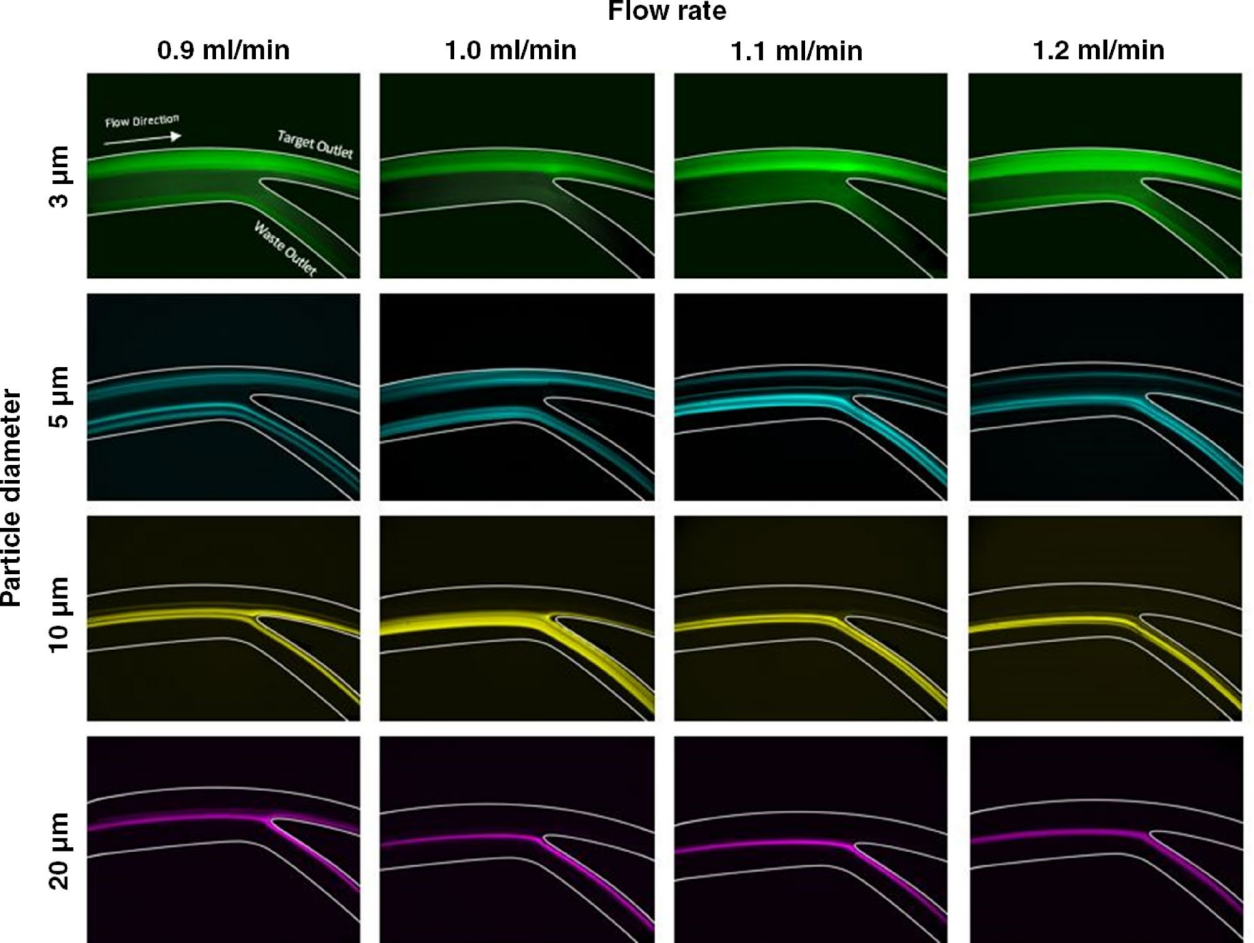
Focusing behaviour of tested microparticle sizes (d. 3, 5, 10, and 20 µm) at flow rates of 0.9, 1, 1.1, and 1.2 mL/min.

Before testing our 3D printed microfluidic device with human samples (a suspension of sperm, WBCs, RBCs and K562 cells), the chips performance was characterised with a range of fluorescent particles, sized similarly to the corresponding cells. The sizes used were 3 µm for representing sperm, 5 µm for RBC, 10 µm for WBC, and 20 µm for invasive cancer cells. 3 µm beads have been shown to be more accurately representative of sperm cells in spiral channels than 5 or 7 µm particles^37^. Figure 2 shows the results of each particle size at the various flow rates of 0.9 mL/min, 1 mL/min, 1.1 mL/min and 1.2 mL/min. Each picture is made up of a stack of frames from a video of each flow through, this provides a clear response of all the particle movements. In a spiral microchannel, the interaction of inertial lift (${{\varvec{F}}}_{{\varvec{L}}}$) and Dean drag ${({\varvec{F}}}_{{\varvec{D}}})$ determine the focusing position of cells and particles in a channel cross-section. These two forces are described in Eqs. (1) and (2).

1 $${F}_{L}=\rho \left(\frac{{U}_{max}}{{D}_{h}}\right){C}_{L}{a}^{4}$$

2 $${F}_{D}=5.4\times {10}^{-4}\pi \mu {De}^{1.63}a$$

here, $a$ is the particle diameter, $\rho$ is the density, $\mu$ is the dynamic viscosity, ${D}_{h}$ is the channel hydraulic diameter, ${U}_{max}$ is the maximum velocity, ${{\varvec{C}}}_{{\varvec{L}}}$ is lift coefficient, and $De$ is Dean number which is used to characterize the strength and power of the Dean flow. Within a straight channel, cells are affected by two forces of wall-induced and shear gradient induced lift forces, both of which are constituent of inertial lift force. Initially, shear gradient lift forces push particles toward the channel wall. On the other hand, in the vicinity of the channel wall, wall induced lift forces repel particles from walls to the channel centre and balances the opposing shear gradient lift force to determine the final focusing position of the particles within a straight channel. In channels with curvature or spiral microchannels, another force (Dean drag force) also affects particles, working to further modulate the particle focusing position. Based on Eqs. (1) and (2), cells with different diameters experience different amounts of inertial lift and Dean drag forces and can be isolated from various outlets. Accordingly, larger particles are more affected by inertial lift forces while smaller particles are dominated by Dean drag forces (${F}_{L}\propto {a}^{4}, {F}_{D}\propto a$). Although previous studies show that cells and particles can focus on a channel when $a/{D}_{h}>0.07$, this number must be adjusted for rigid microfluidic channels such as 3D printed microchannels. This is due to the fact that PDMS-made microchannels are prone to inflation at high flow rates whiles rigid microchannels are more stable in this regard. Deformation of channel walls is an important factor determining the accuracy of particle focusing. Through careful analysis of spiral microchannels, we have shown that a spiral microchannel with heights of 30 and 90 and width of 300 µm is able to focus particles as small as 3 µm.

**Figure 3:**
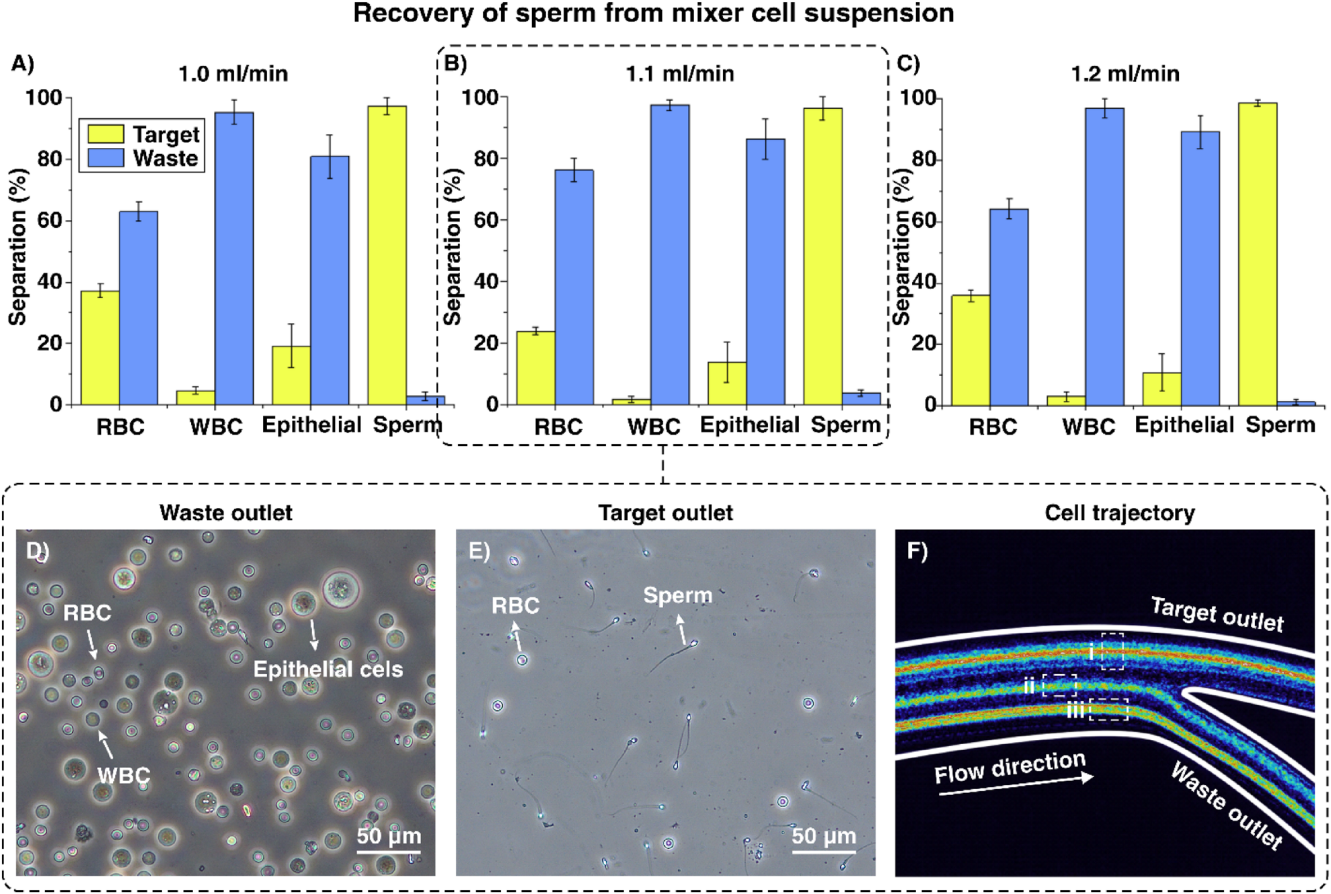
Separation efficiency of cells per outlet at the flow rate of (**A**) 1 mL/min, (**B**) 1.1 mL/min, and (**C**) 1.2 mL/min where N = 10 for peach panel and separation efficiency is presented as the average percentage efficiency ± standard deviation. (**D**, **E**) Side-by-side example brightfield images of collected waste and target fractions collected from microfluidic sorting at 1.1 mL/min respectively. (**F**) Stacked distribution of cell focusing bands at 1.1 mL/min where (i) depicts the combined focusing of sperm cells and RBCs through the target outlet, (ii) depicts the presence of WBCs moving towards the waste outlet, and (iii) depicts a more concentrated band of RBCs exiting through the waste outlet.

Figure 2 reveals that 3 µm particles are consistently close to the outer wall, 5 µm particles occupy a double-focusing band, and 10 and 20 µm particles are focused at the channel inner wall. The flow rate of 1 mL/min was found to be the most optimal flow rate for focusing the sperm sized representative particles through the top channel, closely followed by the flow rate of 1.1 mL/min. With the other three particle sizes, flow rates of 1.1 mL/min and 1.2 mL/min were the most effective at focusing the particles through the waste outlet. While 1.2 mL/min may be a slightly more effective for reducing the collateral cells, higher flow rates put more strain on the channel. Therefore, we decided that the most effective flowrate, for segregating the 3 µm particles from the 5, 10 and 20 µm particles, was 1.1 mL/min. However, due to the non-spherical geometries of both sperm and RBCs, the inherent differences between cell and particle densities, and the heterogeneity in EC, WBC, and K562 cell populations, the same flow rates were re-evaluated for mixed cell suspensions. Solutions of sperm and collateral cells (RBCS, WBCs, and ECs) commonly found in a clinical testicular tissues extraction were tested at various flow rates (Fig. 3).

### Recovery of sperm from mixed cells suspension

Figure 3A–C show the separation efficiency of cells at 1, 1.1 and 1.2 mL/min. While all three successfully separated WBC and sperm, the flow rate of 1.1 mL/min proved the best performance for RBC separation due to a new double band focusing phenomena not seen in PDMS spiral microfluidics (> 75% removal). This is likely occurring due to the conflicting inertial and viscous forces present in a hard chip around cells of RBC size. Sperm on the other hand, exhibited tight focusing on an all tested flow rates (> 96% sperm recovery). WBCs also had high average separation while EC separation gradually improved with increasing flow rate. EC populations typically fell into the size range of 12–22 µm and achieved 81%, 86% and 89% separation at 1, 1.1 and 1.2 mL/min respectively. While the flow rate of 1.2 mL/min provided a slightly higher percentage of sperm and ECs in the target outlet than the flow rate of 1.1 mL/min, it also included slightly more WBCs and much more RBCs. From this, we can deduce that the optimal flow rate for separating sperm from collateral cells in this microfluidic device is 1.1 mL/min, consistent with the findings from the focusing behaviour of microparticles tested within the channel. Figure 3D and E show the cell populations present in the waste and target fractions, respectively. Figure 3F illustrates a stacked recording of cell focusing and separation of cells through the outlets of the microfluidic device. Figure 3F (i) is a mixed band showing the overlay of sperm and RBC focusing while (ii) shows a combination of WBCs and ECs, and iii) shows RBC focusing streams, respectively. While the intensity of the focusing band heat map remained largely unchanged between different flow rates in sperm and WBCs plus ECs, the intensity of the RBC focusing band was dependent on the flow rates which correlated to the changes in cell recovery. As illustrated between graphs A–C in Fig. 3, the change in flow rates affected the distribution of RBC focusing with a flow rate of 1.1 mL/min yielding the best level of RBC removal. RBCs are typically the most numerous background cell type in biopsied tissue, and a > 75% reduction in RBCs facilitates a significant decrease in the background contamination to the point where sperm may become visible.

Inertial microfluidics has been used to isolate sperm from background cell populations previously, with mixed success. Son *et al. *demonstrated sperm recovery from WBCs and then RBCs in a polydimethylsiloxane (PDMS) spiral chip, simulating a leukospermic sample^38,39^. However, the recovery of sperm in a mixed cell suspension with tight and distinguishable focusing streams has remained elusive. Furthermore, to date no study has demonstrated the separation of heterogenous epithelial cells from a mixed sperm in suspension. More recently Nepal et al.demonstrated sperm isolation from a collection of RBCs and WBCs through a four outlet PDMS spiral^40^. While the sperm, RBC, and WBC isolation was reportedly 90%, 89%, and 74% respectively, the recovered cell suspensions were split between four outlets in mixed ratios. The study did not assess the impact of microfluidic separation on essential sperm metrics.

Due to the relatively large size different between sperm, WBCs, and ECs, these results indicate microfluidics as a potentially useful candidate for the removal of WBCs from pyospermic samples in semen, which is defined by the presence of greater than 1 × 10^6^ WBCs per mL of semen^41^. To test the effectiveness of our chip for this case we spiked known concentrations of WBCs (0.2, 0.5 and 1 × 10^6^ WBCs/mL) into raw semen and attempted sperm purification. While diluted semen samples demonstrated non-Newtonian behaviour and no significant cell separation, briefly centrifuged semen samples buffered with SR media demonstrated a consistently high separation efficiency at a flow rate of 1.1 mL/min (See ESI video). Recent studies on the impacts and treatment of pyospermia have reported WBC concentrations as low as 0.1 × 10^6^ WBCs/mL can significantly affect sperm function through the generation of reactive oxygen species (ROS)^42,43^. This makes our microfluidic design suitable for processing pyospermic samples from ejaculated semen in order to retrieve sperm cells quickly before ROS can negatively affect sperm vitality or induce DNA fragmentation.

**Figure 4:**
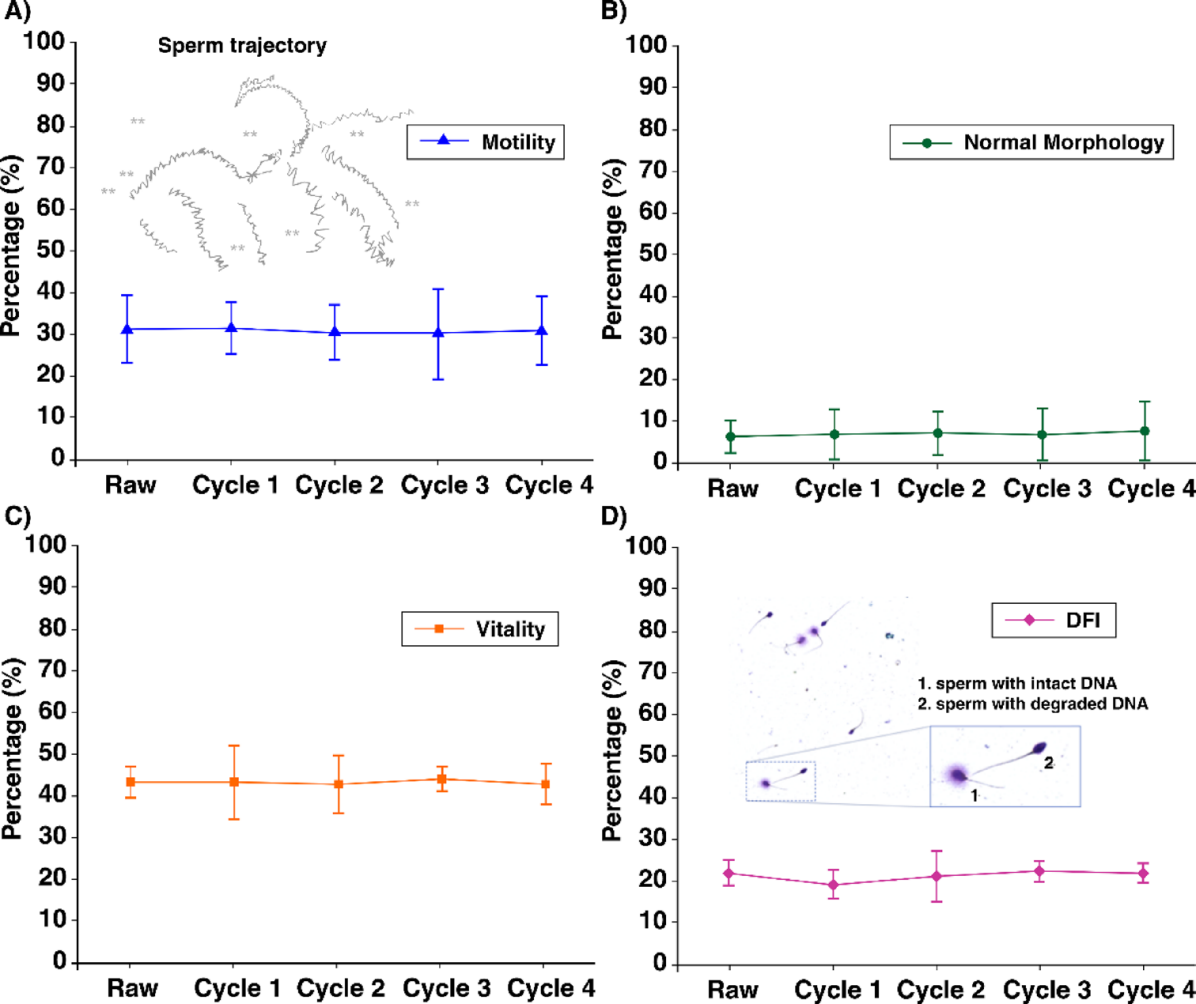
The influence of multiple separation cycles on (**A**) motility, (**B**) morphology, (**C**) vitality and (**D**) DFI of the retrived sperm as compared with the initial raw sample. (**D**) (i) Example images of SCD assayed sperm with sperm 1. Showing intact DNA and sperm 2. Showing degraded DNA. N = 3 for each panel.

While this device serves to reduce the time that sperm are exposed during this separation process, it is also important to ensure that the device itself does not adversely affect sperm cell health. According to Ouitrakul et al., sperm vitality and motility significantly decreases after 2 h post ejaculation. After 2 h, motility reduces by 7% and the vitality reduces by 5%^16^. DFI is strongly correlated to vitality and motility; low vitality infers a high DFI and vice versa^44^. As extraction procedures can take several hours to complete, the continued negative effect on the sperm can be quite detrimental to the viability, motility and DNA fragmentation^12^. Considering this, four experiments were conducted to observe the effect the microfluidic chip has on the motility, morphology, vitality and DFI through multiple microfluidic separation runs (Cycles) (Fig. 4). It is worth noting that a single sperm spends less than 0.25 s in the chip during separation.

As can be seen, Fig. 4 demonstrates consistent averages amongst the sperm parameters indicating no damage induced by the microfluidic device even when the same sample was passed through the device multiple times. It is also worth noting that there is no improvement since we are facilitating sperm recovery, not selection. The sample used in these experiments are designed to see the potential of the chips, though the clinical case of mTESE samples may have as little as 5–20 sperm present. mTESE sperm are also non-motile and therefore ICSI must be used. However, in the case of pyospermic sperm samples where motile sperms are present, it is important to remove sperm from leukospermic contamination whilst also preserving the motility, morphology and genetic integrity of sperm for freezing, conventional IVF, or ICSI. With the use of this microfluidic device for sperm retrieval, the length of time for this process is reduced from 2 to 3 h to less than 5 min. This represents an automated 36-fold improvement in operation time over manual sperm identification, eliminating much of the risks associated with human error and fatigue. This is an important benefit since rates of sperm recovery diminish with time of microscopic examination of biopsied testicular tissues^12^; Human fatigue, inexperience and oversight can cause a negative effect on the efficiency of the selection process, particularly since background cells such as RBCs, WBCs and cancer cells outnumber sperm by orders of magnitude^14,45^. What’s more, the longer the procedure, the more prone an individual is to human error^14^. Consequently, it is essential that a consistent, autonomous, and more time-conscious procedure is developed to improve the sperm recovery process. Microfluidic sperm isolation helps negate the risk of any time related degenerative effects sperm may encounter during conventional microscopy-based and highly manual selection approaches. The results also demonstrate that this is achieved in a manner that does not significantly harm the sperm cells present in the population. This is extremely important in the case of sperm excised directly from the testicles where normal sperm parameters may be inherently compromised, or sperm production levels are already reduced.

### Isolation of sperm from leukaemia

To further investigate the usefulness of this platform in cases where blood borne malignancies such as leukaemia may have penetrated the site of spermatogenesis, we performed the separation of K562 leukemic cells from sperm. The preservation of fertility in these cases is often of paramount concern for sub-fertile adult patients who require testicular sperm retrieval prior to cancer therapy, and for peri-pubertal patients looking to preserve their fertility for the future.

**Figure 5:**
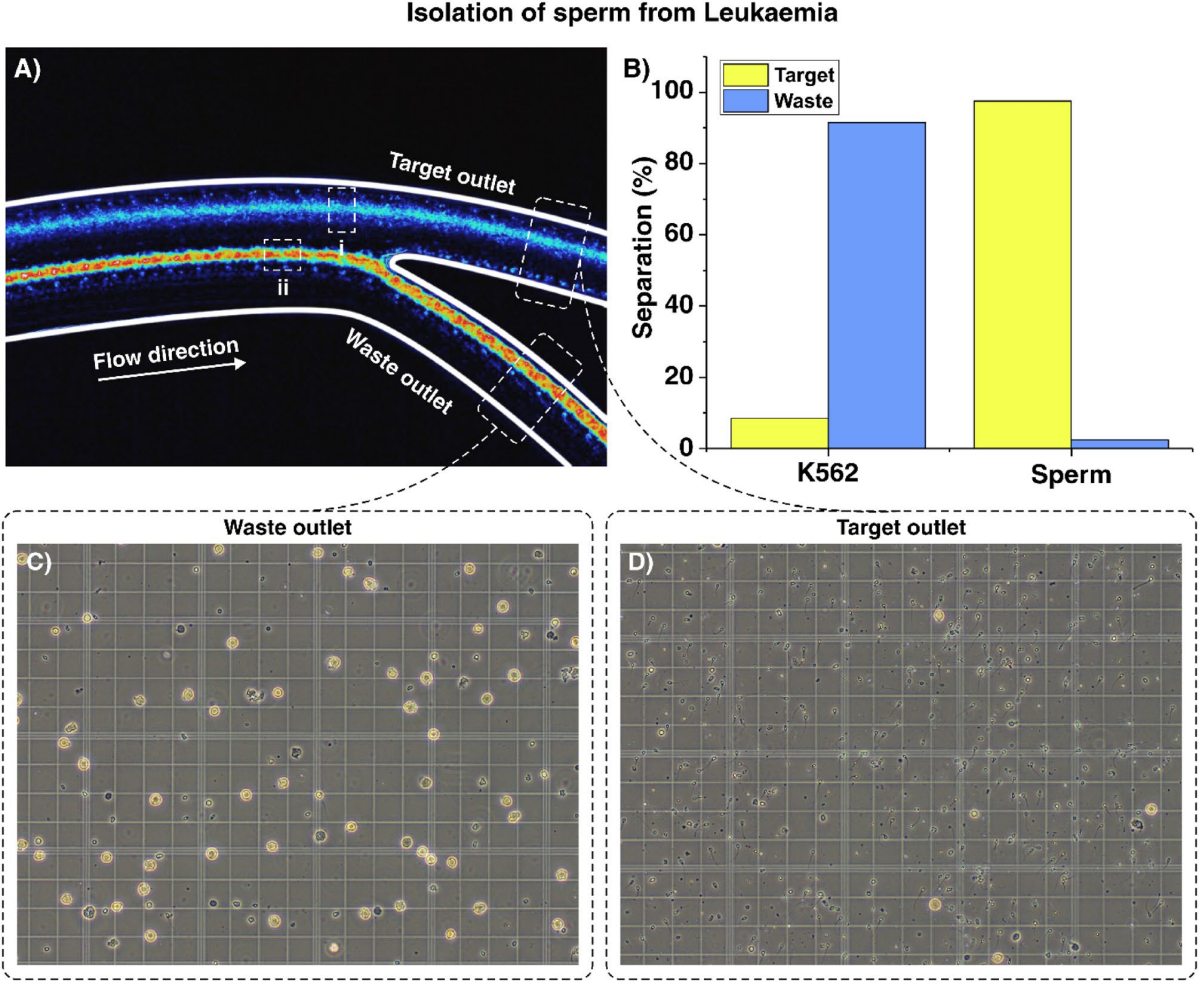
Separation of sperm from K562 leukemic cells (**A**) high speed video stack of outlet focusing where (i) shows the tight focusing band of sperm (flow rate of 1.1 mL/min) and (ii) shows the focusing band of K562. (**B**) Separation efficiency where N = 5 and separation efficiency is presented as the average percentage efficiency ± standard deviation. (**C**, **D**) Waste and target fractions, respectively.

K562 cells separate well (> 95%) from sperm in high concentrations, as can be seen by the stacked image in Fig. 5A and from the graph in Fig. 5B. Figure 5A (i) which shows the tight focusing band of sperm (flow rate of 1.1 mL/min) showing almost no crossover into the waste outlet. Figure 5A (ii) shows the focusing band of K562 leukemic cells with a dense focusing band at the same flow rate. Despite the heterogeneity of cultured cell lines compared to synthetic beads, this focusing behaviour is consistent with the 20 µm focusing behaviour observed in experiments with fluorescent particles. Figure 5C,D provide images of recovered cell fractions from the waste and target outlets, respectively. This is the first microfluidic attempt to separate leukemic cells from sperm that can achieve stable and tight band focusing with such a high efficiency. The results obtained from K562, WBC, EC, and RBC separation from sperm are encouraging and warrant testing on clinical testicular biopsied samples for use in fertility preservation.

## Conclusions

Non obstructive Azoospermia is an infertility condition affecting 10% of infertile men. The processes in place to recover sperm from men who undergo surgery from this condition are antiquated and potentially detrimental to the quality of the sperm found. In this study, we have successfully demonstrated the application of 3D printed microfluidic hard chips to recover sperm from mixed cell suspensions with a > 96% recovery rate. Our method drastically improves the recovery time of human sperm from several hours of manual observation to minutes of automated sperm recovery, representing a 36-fold improvement in standardised sample processing time. Microfluidic recovery of sperm not only reduces clinical labour but also decreases the risk of human error and fatigue during sample processing of biopsied tissue. We have also shown that the application of microfluidics has no detrimental effect on the motility, vitality, morphology or DFI of the sperm collected when compared to the raw sample. We also observed novel double-band focusing behaviour of RBCs and 5 µm particles only present in hard microfluidic spirals. Applying this approach, as a more automated and time effective approach, could greatly improve the current processing procedure of mTESE, MESA, TESE, and TESA samples, and by extension fertility outcomes for such sever forms of male infertility that warrant the surgery.

## Supplementary Information

Supplementary Video 1.

Supplementary Information 1.
