# Supplementary material for: A microfluidic approach to rapid sperm recovery from heterogeneous cell suspensions
Source: Sci Rep. 2021 Apr 12;11:7917. doi: 10.1038/s41598-021-87046-9 (PMC8042033; doi:10.1038/s41598-021-87046-9)
Supplement: Supplementary file 2 — Supplementary Information 1. [file 41598_2021_87046_MOESM2_ESM.docx]

Electronic supplementary information

**A Microfluidic Approach to Rapid Sperm Recovery from Heterogeneous Cell Suspensions**

Steven A. Vasilescu^1^, Shayan Khorsandi^1^, Lin ding ^1^, Sajad Razavi Bazaz^1^, Reza Nosrati^2^, Debra Gook^4^, Majid Ebrahimi Warkiani^1,3^*

Herein is a detailed description of aforementioned methods and details from the main text. This supplementary file includes:

- Information on Materials used for 3D printing fabrication.
- Image of microfluidic setup during fluorescent particle experiments and mixed cell suspension use.

**Materials and resin used for microfabrication**

The selection of appropriate raw materials for microchannel fabrication has been a subject of interest for many years. Conventionally, microfluidic devices are fabricated from glass, silicon, elastomers, and even metals. While elastomers and glass are ideal for fluid imaging due to their transparency, they are not amenable to upscaled production. Elastomers are also gas permeable and possess the potential to be penetrated by small molecules and particles such as nanoparticles. Thermoplastics on the other hand are generally considered to be gas impermeable and allow for quick turnover times between significant design changes.

The resin used in this study (BV-007) is commercially available from Creative CADworks and is which was developed specifically with microfluidics in mind. The two major components of this photopolymer resin are oligomers and acrylate monomers. Acrylate monomers and oligomers primarily contain vinyl; however, in addition to this, photoinitiator components are added to the acrylate. The viscosity and density of the liquid properties of the BV-007 resin are previously reported as <100 cps at 28 ºC and 1.04 g/ml at 25 ºC respectively. Furthermore, the hardness of the resin is reported to be 75 Shore D at 25 ºC. Considering that the viscosity of the resin uncured is somewhat similar to that of water, removal of excess resin following printing is not difficult.


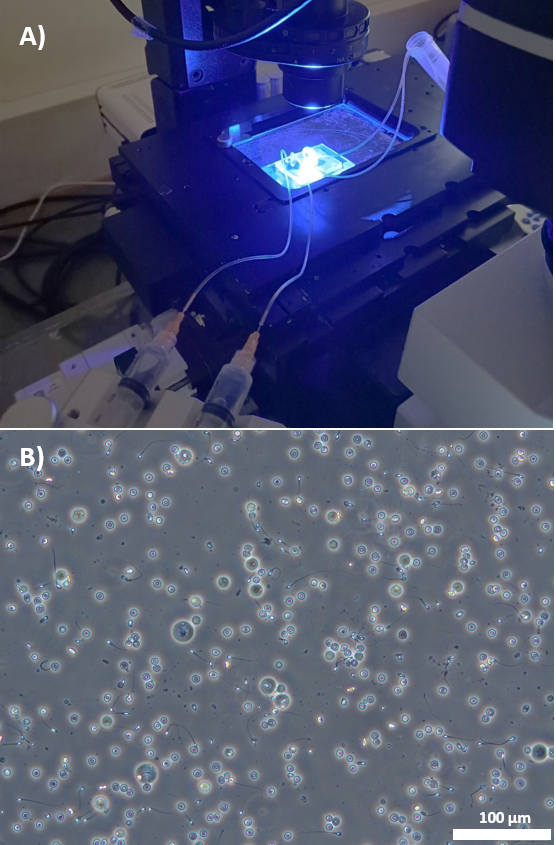


Figure 1. A) Microfluidic setup of 30.90.300 chip during fluorescent particle B) mixed cell suspension of sperm, RBCs, WBCs, and ECs of various sizes before microfluidic cell separation.
